# Dipeptidyl peptidase-4 inhibitors and cardiovascular events in patients with type 2 diabetes, without cardiovascular or renal disease

**DOI:** 10.1371/journal.pone.0240141

**Published:** 2020-10-15

**Authors:** Sheriza N. Baksh, Jodi B. Segal, Mara McAdams-DeMarco, Rita R. Kalyani, G. Caleb Alexander, Stephan Ehrhardt

**Affiliations:** 1 Department of Epidemiology, Johns Hopkins Bloomberg School of Public Health, Baltimore, MD, United States of America; 2 Center for Drug Safety and Effectiveness, Johns Hopkins University, Baltimore, MD, United States of America; 3 Department of Health Policy and Management, Johns Hopkins Bloomberg School of Public Health, Baltimore, MD, United States of America; 4 Center for Health Services and Outcomes Research, Johns Hopkins University, Baltimore, MD, United States of America; 5 Division of General Internal Medicine, Department of Medicine, Johns Hopkins Medicine, Baltimore, MD, United States of America; 6 Division of Endocrinology, Diabetes & Metabolism, Department of Medicine, Johns Hopkins University School of Medicine, Baltimore, MD, United States of America; University of Messina, ITALY

## Abstract

**Background:**

Cardiovascular safety of dipeptidyl peptidase-IV inhibitors (DPP-4i) in patients without cardiovascular or renal disease, a majority of newly diagnosed patients with type 2 diabetes often excluded from clinical trials on this association, is poorly understood. Thus, we investigate the risk of major adverse cardiovascular events (MACE) associated with DPP-4i in low-risk patients with diabetes

**Methods:**

Using a new-user retrospective cohort derived from IBM MarketScan Commercial Claims and Encounters (2010–2015), we identified patients aged 35–65 with type 2 diabetes, without cardiovascular or renal disease, initiating DPP-4i, sulfonylureas, or metformin. Primary composite outcome of time to first MACE was defined as the first of any of the following: myocardial infarction, cardiac arrest, coronary artery bypass graft, coronary angioplasty, heart failure, and stroke. Secondary outcomes were time to first heart failure, acute myocardial infarction, and stroke. We compared outcomes for DPP-4i versus sulfonylurea and DPP-4i versus metformin using propensity score weighted Cox proportional hazards, adjusting for demographics, baseline comorbidities, concomitant medications, and cumulative exposure.

**Results:**

Of 445,701 individuals, 236,431 (53.0%) were male, median age was 51 (interquartile range: [44, 57]), 30,267 (6.79%) initiated DPP-4i, 52,138 (11.70%) initiated sulfonylureas, and 367,908 (82.55%) initiated metformin. After adjustment, DPP-4i was associated with lower risk of MACE than sulfonylurea (adjusted hazard ratio (aHR) = 0.87; 95% confidence interval (CI): 0.78–0.98), and similar risk to metformin (aHR = 1.07; 95% CI: 0.97–1.18). Risk for acute myocardial infarction (aHR = 0.70; 95% CI: 0.51–0.96), stroke (aHR = 0.57; 95% CI: 0.41–0.79), and heart failure (aHR = 0.57; 95% CI: 0.41–0.79) with DPP-4i was lower compared to sulfonylureas.

**Conclusion:**

Our findings show that for this cohort of low-risk patients newly treated for type 2 diabetes, DPP-4i exhibited 13% lower risk for MACE compared to sulfonylureas and similar risk for MACE compared to metformin, suggesting DPP-4i is a low cardiovascular risk option for low-risk patients initiating antihyperglycemic treatment.

## Introduction

Type 2 diabetes is prevalent in 9.4% of the United States population, affecting individuals of different races, ages, and socio-economic backgrounds [1]. The long-term micro- and macro-vascular complications of diabetes compound the public health impact of the disease. Additionally, diabetes often presents in patients with multiple comorbidities, many affecting the cardiovascular system [2]. Of the 7.2 million hospital discharges for patients with diabetes in 2014, 1.5 million were for cardiovascular events [1]. Mitigating the risk for major adverse cardiovascular events (MACE) involves a nuanced understanding of a patient’s blood glucose levels, comorbidities, vascular complications, and medication regimen.

Further complicating diabetes management, clinical trials and retrospective cohort studies have linked some drug classes, such as thiazolidinediones [3] and sulfonylureas [4] to an increased risk of MACE. Determining whether these associations are due to underlying cardiovascular disease or to adverse reactions from a particular drug remains a challenge for regulators and practitioners [5]. One newer class of oral antihyperglycemic agents, dipeptidyl peptidase-IV inhibitors (DPP-4i), has elicited increased reports of hospitalization for heart failure to the United States Food and Drug Administration Adverse Event Reporting System [6, 7]. They were initially believed to be cardio-protective in pre-market clinical pharmacology studies [8]. However, postmarketing clinical trials [9–11] and retrospective, insurance claims-based cohort studies [12–15] have reported inconsistent data regarding the relationship of DPP-4i to hospitalization for heart failure. Additionally, the clinical trials have focused primarily on individuals with established cardiovascular disease. While most insurance claims-based cohort studies have primarily focused on all diabetic patients exposed to DPP-4i irrespective of established cardiovascular or renal disease, one study from Taiwan suggests lower risk of MACE in non-CKD patients treated with DPP-4i compared to those not treated with DPP-4i [15]. Importantly, the majority of new users of oral antihyperglycemic agents do not have established cardiovascular or renal disease. We sought to better understand cardiovascular safety of DPP-4i for this population in a real-world setting.

Using a nationwide commercial claims database, we investigated the association between DPP-4i therapy and MACE. We restricted the study population to those aged 35–65 without diagnosed cardiovascular or renal disease and compared time to first MACE for new users of DPP-4i versus new users of metformin, and for new users of DPP-4i versus new users of sulfonylurea. Additionally, we compared incidence and time to event for hospitalization for heart failure, stroke, and acute myocardial infarction.

## Methods

### Study design and data source

We conducted a retrospective cohort study of commercially insured patients, using IBM MarketScan Commercial Claims and Encounters data from January 2010 through December 2015. This database captures individual-level linked patient claims and encounter data for approximately 25 million individuals annually. The de-identified data contains patient demographics, inpatient and outpatient services, and prescription drug claims.

### Study population

We identified patients with type 2 diabetes as those with at least one prescription for an oral antihyperglycemic agent and either hemoglobin A1c greater than 6.5% (48 mmol/mol) twice, fasting glucose greater than 126 mg/dL twice on different days, random glucose > 200 mg/dL twice on different days, one inpatient diagnosis (International Classification of Diseases, 9^th^ /10^th^ Revisions (ICD-9(10)): 250x(E11.9), 357.2(E11.42), 366.41(E11.36), 362.01–362.07(E11.3*)), or outpatient diagnosis (ICD-9(10): 250x(E11.9), 366.41(E11.36), 362.01–362.07(E11.3*)) twice on different days. Due to the limitations of distinguishing between patients with type 1 diabetes and those with type 2 diabetes through ICD-9/10 codes, we only included patients above the age of 35 to increase our specificity for patients with type 2 diabetes and minimize the misclassification of patients with type 1 diabetes as those with type 2 diabetes. We included patients if they received at least one prescription for an FDA-approved DPP-4i, sulfonylurea, or metformin (**S1 Table**). The index date for follow-up was assigned as the date of first filled prescription for one of these products. The baseline period was defined as the six-month period preceding this.

We excluded individuals if they met any of the following criteria: 1) less than six months of continuous medical and prescription enrollment, 2) less than 12-weeks of continuous exposure to exposure group drugs, 3) insulin use during baseline period, 4) treatment with other oral or injectable antihyperglycemic agents in baseline period, 5) cardiovascular disease or renal disease in baseline, and 6) missing age or sex information. We followed patients until the first of the following events: 1) first MACE, 2) end of continuous medical or prescription enrollment, 3) switch in antihyperglycemic agent treatment or addition of another antihyperglycemic agent, 4) 14-days after the last date of exposure to exposure group drug, or 5) study end date of December 31, 2015.

We identified individuals with established cardiovascular disease through ICD-9/10 codes for myocardial infarction, complete atrioventricular block, cardiogenic shock, coronary artery disease, chronic heart failure, stroke, cerebral infarction, atrial fibrillation, or coronary artery bypass graft in the six-month period before baseline. We also defined renal disease through ICD-9/10 codes for chronic kidney disease or acute renal failure in the six-month period before baseline.

### Definition of exposure

The three exposure groups consisted of new users of DPP-4i, sulfonylurea, and metformin, respectively (**S1 Table**). We created an indicator variable for cumulative exposure, defined as days exposed to the exposure group drug. In the event of an individual initiating more than one of these drug classes at baseline, they were assigned to all relevant exposure groups. We used a negative control (metformin) and positive control (sulfonylurea) in this study for two reasons: 1) the population of new users of DPP-4i and sulfonylurea are more similar than DPP-4i and metformin users; however, sulfonylurea users generally have lower metabolic control, and sulfonylurea carries cardiovascular risk; 2) metformin carries low cardiovascular risk; however, new users of metformin have less severe diabetes.

### Definition of outcomes

We defined our primary composite outcome of MACE as the first of any of the following events: myocardial infarction, cardiac arrest, coronary artery bypass graft, coronary angioplasty, heart failure, and stroke. Myocardial infarction [16, 17], cardiac arrest [18], coronary artery bypass graft [16], coronary angioplasty [16], heart failure [19], and stroke [20] were identified through validated ICD-9/10 algorithms. For conditions/procedures without validated ICD-10 algorithms, we deferred to the Chronic Conditions Warehouse [21]. We excluded all-cause mortality from the primary composite outcome due to dataset limitations. Secondary outcomes were acute myocardial infarction, stroke, and hospitalization for heart failure.

### Definition of covariates

We assessed possible confounding due to individual demographics, concomitant medications, and comorbidities. We conducted literature searches of similar studies [17, 22–24], consulted clinical guidance [25–28], and regulatory documents [29] to identify covariates of interest, and we created indicator variables for individuals’ age and sex. We accounted for comorbidities such as hypertension, asthma, peripheral vascular disease, and neuropathy using the Clinical Classification Software [30]. Additionally, we identified the use of concomitant medication by their National Drug Codes. Finally, we categorized individuals based on their disease severity using the Adjusted Diabetes Comorbidities Severity Index (aDCSI) [31–33]. A full list of covariates used in the analysis is contained in **S2 Table**.

### Propensity score

We first identified all available covariates without an association to the exposure that were associated to the primary outcome in order to increase precision and reduce the potential bias from unmeasured variables [34]. Next, we used the Toolkit for Weighting and Analysis of Nonequivalent Groups (*twang)* package for R developed by the RAND Corporation to compute the propensity scores and associated weights used in the analysis to balance the covariates between exposure groups. This allowed for propensity scores estimation in the presence of multiple exposure groups. Using generalized boosted regression models, we optimized the selection of covariates for the propensity score calculation. We used the standardized mean difference (SMD) to measure the balance of covariates before and after weighting. Propensity score weighting reduced the SMD from a maximum of 0.15 to less than 0.01. We then used the average treatment effect on the treated (ATT) propensity score weights to estimate the treatment effect of DPP-4i.

### Statistical analysis

We used chi-square statistics for categorical covariates and the Kruskal-Wallis test for continuous covariates to compare differences at baseline between new users of DPP-4i, sulfonylurea, and metformin. Next, we used exact Poisson tests to compute the incidence rate differences for the primary and secondary outcomes between new users of DPP-4i and those of sulfonylurea and metformin, respectively. Additionally, we checked for differences in time to first MACE distributions using a Kolmogorov-Smirnov test. We calculated propensity score weighted crude and adjusted Cox proportional hazards for the association between new use of DPP-4i and the primary and secondary outcomes compared to new use of sulfonylureas and metformin. We included indicators for age, sex, baseline comorbidities, and concomitant medication in the adjusted models (**S2 Table**). We plotted the scaled Schoenfeld residuals to check for covariates that violated the proportional hazards assumption and stratified the Cox proportional hazards model by covariates that violated the proportional hazards assumption. Finally, we included natural regression spline terms with knots at 180-, 365-, and 540-days to account for changes in the underlying hazard function with increasing cumulative exposure, which was defined as the total days exposed to the exposure group drug [35]. All analyses were conducted in R, version 3.3.3.

### Sensitivity analyses

To check the robustness of our results, we first assessed sensitivity of our results to the latency of the period after drug discontinuation. We lagged this period for 7-, 14-, and 30-days after the last day of exposure to drug. Next, we recalculated the primary analysis without individuals exposed to more than one exposure group to determine whether our results were sensitive to the inclusion of those individuals.

The study was exempted from review by a Johns Hopkins Institutional Review Board.

## Results

### Patient inclusion and characteristics

**Fig 1:**
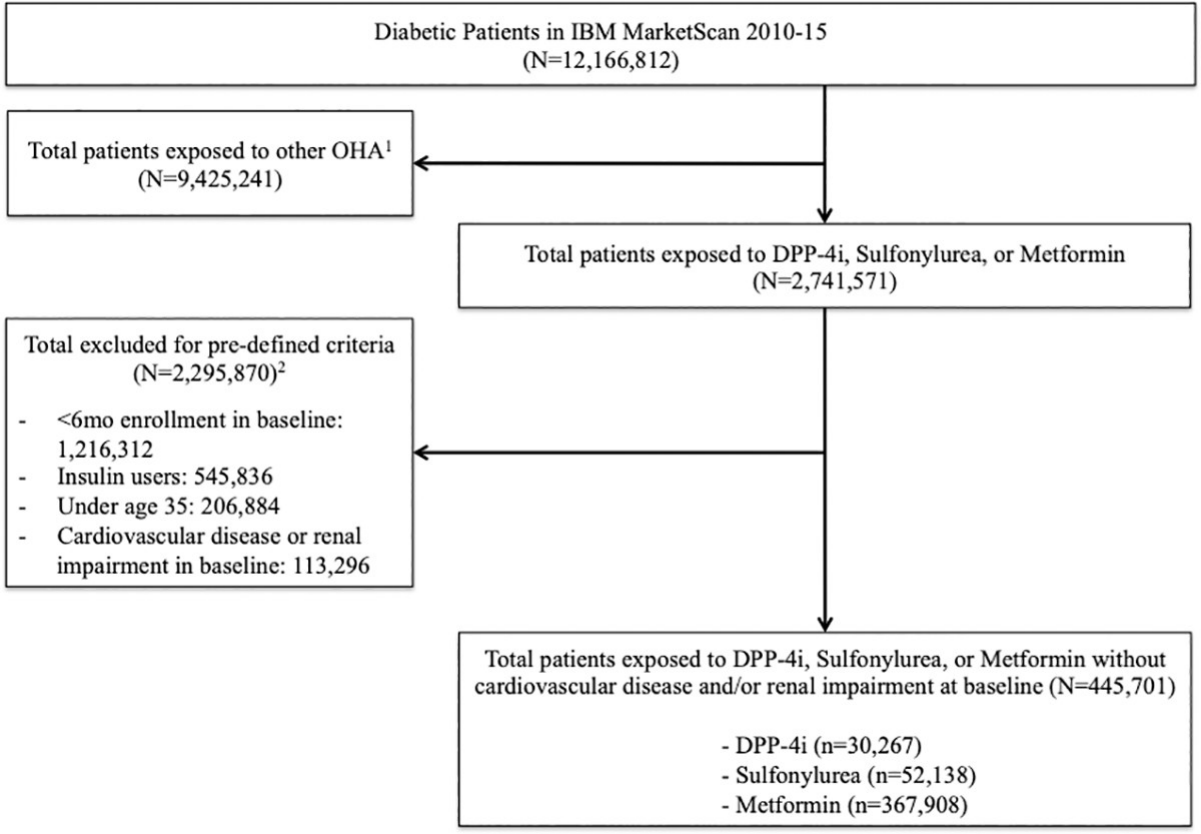
Cohort derivation and sample attrition. Diagram depicting the cohort derivation and sample attrition after the application of inclusion and exclusion criteria. ^1^ OHA = oral antihyperglycemic agents^2^ Subcategories are not mutually exclusive.

We first identified 12,166,812 individuals with diabetes from January 2010-December 2015 through commercial claims. More individuals on DPP-4i (41.51%) and sulfonylureas (44.52%) were aged 55–65 compared to those on metformin (36.37%). After applying the inclusion and exclusion criteria above, the study population consisted of 445,701 individuals. There were 30,267(6.79%) new users of DPP-4i, 52,138(11.70%) who initiated sulfonylureas, and 367,908 (82.55%) who started metformin (Fig 1). Less than one percent of included individuals were new users of both DPP-4i and metformin, and 17,070 (3.83%) were new users of both sulfonylureas and metformin.

Individuals in each exposure group differed in their baseline characteristics (**Table 1**). There were more male new users of sulfonylureas (58.19%) than DPP-4i (56.86%) or metformin (51.34%). Most individuals in each group were exposed for 12 months or less (DPP-4i: 77.94%; sulfonylurea: 76.27%; metformin: 72.41%). Many individuals in this study cohort experienced vascular complications of diabetes. New users of DPP-4i had neuropathy (7.53%), retinopathy (5.48%), and peripheral vascular disease (2.79%) in the baseline period. Among new users of sulfonylurea, 6.93% had neuropathy, 5.04% had retinopathy, and 2.17% had peripheral vascular disease. Metformin users had the lowest proportion of peripheral vascular disease (1.87%), retinopathy (3.50%), and nephropathy (0.54%). Additionally, a higher percentage of DPP-4i initiators (22.3%) used angiotensin II receptor blockers in baseline compared to sulfonylurea (16.2%) and metformin (16.5%) initiators. Statin use was also higher in DPP-4i initiators (42.5%) than sulfonylurea (37.5%) and metformin (37.9%). Differences in baseline characteristics between exposure groups were diminished after propensity score weighting.

**Table 1:** Baseline demographics and medical characteristics by exposure group.

	DPP-4i (n = 30,267)		Sulfonylureas (n = 52,138)		Biguanides (n = 367,908)	
	N	%	N	%	N	%
***Demographics***						
Male	17,209	56.86	30,340	58.19	188,882	51.34
Age						
35–44	5,562	18.38	9,578	18.37	82,260	22.36
45–54	12,140	40.11	19,348	37.11	151,847	41.27
55–64	12,565	41.51	23,212	44.52	133,801	36.37
Location (Region)						
Northeast	5,829	19.26	7,358	14.11	56,186	15.27
North Central	5,614	18.55	10,635	20.40	79,433	21.59
South	14,926	49.31	25,108	48.16	160,626	43.66
West	3,391	11.20	8,203	15.73	65,961	17.93
Unknown	507	1.68	834	1.60	5,702	1.55
***Cumulative Exposure***						
<6 months	13,110	43.31	24,428	46.85	141,469	38.45
6–12 months	10,481	34.63	15,339	29.42	124,943	33.96
12–18 months	3,456	11.42	5,971	11.45	49,787	13.53
>18 months	3,220	10.64	6,400	12.28	51,709	14.05
***Comorbidities in Baseline***						
Asthma	1,823	6.02	2,578	4.94	25,700	6.99
Peripheral Vascular Disease	845	2.79	1,134	2.17	6,886	1.87
Ischemic heart disease	1,201	3.97	1,861	3.57	11,364	3.09
Hypertension	18,907	62.47	30,789	59.05	215,790	58.65
Retinopathy	1,659	5.48	2,630	5.04	12,870	3.50
Eye disease	8,009	26.46	11,759	22.55	86,274	23.45
Renal disease	7,390	24.42	11,008	21.11	84,100	22.86
Atrial fibrillation	2,070	6.84	2,988	5.73	26,749	7.27
Neuropathy	2,280	7.53	3,612	6.93	27,841	7.57
Nephropathy	279	0.92	589	1.13	2,000	0.54
aDCSI Score						
0	30,115	99.50	51,845	99.44	366,471	99.61
1+	152	0.50	293	0.56	1,437	0.39
***Dual Exposures***						
Sulfonylureas	--	--	--	--	17,070	4.64
Metformin	2,303	7.61	17,070	32.74	--	--
DPP-4 inhibitors	--	--	--	--	2,303	0.63
***Concomitant Medications at Baseline***						
ACE inhibitors	5,856	19.35	11,255	21.59	84,735	23.03
angiotensin II receptor blockers	6,746	22.29	8,467	16.24	60,645	16.48
antidepressants	4,648	15.36	6,933	13.30	71,046	19.31
Antiplatelets	3,609	11.92	5,353	10.27	46,484	12.63
Asthma medication	819	2.71	1,225	2.35	10,656	2.90
α-Glucosidase inhibitors	58	0.19	80	0.15	184	0.05
benzodiazepines	3,195	10.56	4,844	9.29	46,310	12.59
beta blockers	4,165	13.76	7,640	14.65	57,142	15.53
bile acid sequestrants	526	1.74	644	1.24	4,081	1.11
blood thinners and anticoagulants	538	1.78	968	1.86	7,207	1.96
calcium channel blockers	2,876	9.50	5,298	10.16	35,658	9.69
cardioselective beta blockers	1,405	4.64	2,229	4.28	14,101	3.83
diuretics	4,399	14.53	7,324	14.05	66,953	18.20
cholinergics	12	0.04	16	0.03	184	0.05
hormone replacement therapy	923	3.05	1,100	2.11	14,562	3.96
fibrates	2,668	8.81	3,462	6.64	23,955	6.51
niacin	607	2.01	905	1.74	6,533	1.78
nitrates	217	0.72	482	0.92	2,975	0.81
NSAIDs	5,844	19.31	8,935	17.14	83,764	22.77
bronchodilators	3,123	10.32	4,195	8.05	43,808	11.91
inhaled steroids	4,830	15.96	6,603	12.66	70,760	19.23
oral corticosteroids	6,421	21.21	9,127	17.51	90,048	24.48
erythropoietin	6	0.02	22	0.04	22	0.01
ophthalmic drugs	618	2.04	1,000	1.92	6,262	1.70
disease-modifying antirheumatic drugs	543	1.79	882	1.69	6,891	1.87
biologic response modifiers	171	0.56	259	0.50	1,930	0.52
peripheral neuropathic treatments	1,265	4.18	1,474	2.83	14,991	4.07
statins	12,852	42.46	19,534	37.47	139,601	37.94
thiazide diuretics	5,195	17.16	8,895	17.06	79,174	21.52

### Association between treatment and major adverse cardiovascular events

The median follow-up for first occurrence of MACE was 341 days (interquartile range (IQR): 196–580), and there were no significant differences in follow-up time between exposure groups. The absolute difference in incidence rates for the primary composite outcome was statistically significantly greater for DPP-4i (21.45 per 1,000 person-years) compared to metformin (17.61 per 1,000 person-years). DPP-4i was also associated with a lower incidence rate of MACE than sulfonylurea (24.87 per 1,000 person-years) (**Table 2**). This difference was also seen in the secondary outcomes of acute myocardial infarction (DPP-4i: 2.45 per 1,000 person-years vs. sulfonylurea: 3.72 per 1,000 person-years), stroke (DPP-4i: 2.21 per 1,000 person-years vs. sulfonylurea: 4.08 per 1,000 person-years), and heart failure (DPP-4i: 2.21 per 1,000 person-years vs. sulfonylurea: 4.02 per 1,000 person-years). There were no differences in incidence between DPP-4i and metformin for the secondary outcomes.

**Table 2:** Incidence rates of primary composite outcome, acute myocardial infraction, stroke, and heart failure among new users of DPP-4 inhibitors, sulfonylureas, and metformin.

Outcome	DPP-4 Inhibitors	Sulfonylureas	Metformin
**Primary Composite Outcome**^1^**, (n)**	450	910	5,445
Total person years	20,982	36,595	309,151
Rate per 1,000 person years	21.45	24.87	17.61
Median [IQR]^2^ observation time, days	187 [107, 314]	173 [104, 317]	225 [134, 384]
Rate difference^3^ (95% CI)	--	**-3.42 [-5.98, -0.86]**	**3.83 [1.80, 5.87]**
**Acute Myocardial Infarction, (n)**	52	138	791
Total person years	21,236	37,060	312,411
Rate per 1,000 person years	2.45	3.72	2.53
Median [IQR] observation time, days	189 [108, 317]	176 [104, 321]	227 [135, 388]
Rate difference (95% CI)	--	**-1.28 [-2.19, -0.36]**	-0.08 [-0.77, 0.61]
**Stroke, (n)**	47	151	786
Total person years	21,239	37,027	312,438
Rate per 1,000 person years	2.21	4.08	2.52
Median [IQR] observation time, days	189 [108, 317]	175 [104, 321]	227 [135, 388]
Rate difference (95% CI)	--	**-1.87 [-2.77, -0.86]**	-0.07 [-0.76, 0.62]
**Heart Failure, (n)**	47	149	602
Total person years	21,238	37,052	312,548
Rate per 1,000 person Years	2.21	4.02	1.93
Median [IQR] observation time, days	189 [108, 317]	175 [104, 321]	227 [135, 388]
Rate difference (95% CI)	--	**-1.81 [-2.71, -0.90]**	0.52 [-0.16, 1.21]

^1^ Primary composite outcome includes myocardial infarction, cardiac arrest, coronary artery bypass, coronary angioplasty, heart failure, stroke, death

^2^ IQR = interquartile range

^3^ Incidence rate difference per 1,000 person-years

After adjustment for baseline characteristics, introducing spline terms for every six months of cumulative exposure, and propensity score weighting, there was an association between DPP-4i and MACE compared to sulfonylurea and MACE (adjusted hazard ratio (aHR): 0.87, 95% confidence interval (CI): 0.78–0.98). In contrast, there was no difference in risk for MACE with DPP-4i compared to metformin (aHR: 1.07, 95% CI: 0.97–1.18) (**Table 3**).

**Table 3:** Hazard ratios for the association between DPP-4 inhibitor use and primary composite outcome, acute myocardial infraction, stroke, and heart failure compared to Sulfonylureas and metformin.

	Hazard Ratios for DPP-4 Inhibitors Use			
Reference Group	Primary Composite Outcome^3^	Acute Myocardial Infarction	Stroke	Heart Failure
Sulfonylureas				
HR [95% CI]^1^	**0.86 [0.77, 0.97]**	**0.69 [0.50, 0.95]**	**0.54 [0.39, 0.75]**	**0.58 [0.42, 0.81]**
aHR [95% CI]^2^	**0.87 [0.78, 0.98]**	**0.70 [0.51, 0.96]**	**0.57 [0.41, 0.79]**	**0.57 [0.41, 0.79]**
Metformin				
HR [95% CI]^1^	1.08 [0.98, 1.19]	0.91 [0.69, 1.21]	0.80 [0.59, 1.07]	1.05 [0.78, 1.41]
aHR [95% CI]^2^	1.07 [0.97, 1.18]	0.95 [0.72, 1.27]	0.81 [0.60, 1.09]	1.04 [0.77, 1.40]

^1^Propensity score weighting only

^2^Propensity score weighting, spline terms for cumulative exposure, and demographics, comorbidities, and concomitant medications as regressors and stratifiers

^3^Primary composite outcome includes myocardial infarction, cardiac arrest, coronary artery bypass, coronary angioplasty, heart failure, stroke, death

There were also differences in risk seen for the secondary outcomes between exposure groups. After adjustment, there was a lower risk for acute myocardial infarction associated with DPP-4i (aHR: 0.70, 95% CI: 0.51–0.96) compared to sulfonylurea (aHR: 0.95, 95% CI: 0.72–1.27). Risk for stroke was also lower with DPP-4i (aHR: 0.57, 95% CI: 0.41–0.79) compared to sulfonylurea (aHR: 0.81, 95% CI: 0.60–1.09). Finally, DPP-4i (aHR: 0.57, 95% CI: 0.41–0.79) was associated with a lower risk for heart failure compared to sulfonylurea (aHR: 1.04, 95% CI: 0.77–1.40). There were no statistically significant associations between the secondary outcomes and DPP-4i when compared to metformin in the adjusted or unadjusted analyses (**Table 3**).

### Sensitivity analyses

After removing individuals with more than one exposure group, there were 426,328 individuals in the cohort. Analysis results did not qualitatively differ after removal of these individuals (**S3 Table**). Additionally, results were not sensitive to lagging the latency period by 7-days or 30-days after the last dose of exposure for the primary analysis (**S4 Table**).

## Discussion

In this analysis of commercial claims data for patients with diabetes without baseline cardiovascular or renal disease, DPP-4i use was associated with 13% lower risk of MACE compared to sulfonylureas, and similar risk of MACE when compared to metformin. DPP-4i was also associated with decreased risk for acute myocardial infarction, stroke, and heart failure when compared to sulfonylurea. These results contribute to existing data from surveillance of adverse event reports [6], clinical trials [9, 10, 36, 37], and other cohort analyses [12–15] examining the association of DPP-4i and MACE, by focusing on low cardiovascular risk patients with diabetes.

These results align with those of previous clinical trials of cardiovascular safety of DPP-4i. Unlike the three completed clinical trials comparing DPP-4i and placebo [10, 36, 38] however, our study population consisted of individuals with low-risk for MACE in a real-world setting with multiple comorbidities and concomitant medications. Our results confirm that DPP-4i are similar to metformin and potentially safer than sulfonylureas with regards to risk of MACE in low-risk new users. Additionally, our analysis compared DPP-4i to other common first-line therapies, as opposed to assessing it as add-on therapy compared to placebo. The Cardiovascular Outcome Study of Linagliptin vs. Glimepiride in Type 2 Diabetes (CAROLINA) trial in high-risk participants used an active control and found linagliptin to be non-inferior to glimepiride for time to 3-point MACE (HR: 0.98, 95% CI: 0.84–1.14) [37]. The use of a high-risk study population could explain conflicting results, as CAROLINA was underpowered to test for interactions between treatment and underlying cardiovascular risk at baseline. By restricting our population to low-risk patients, we minimized the possibility of the event rates in this study being driven by underlying cardiovascular risk at baseline.

The results of our secondary outcomes contribute to the existing body of evidence in two respects. Our results showing lower risk for stroke with DPP-4i compared to sulfonylureas is consistent with one systematic review of 301 clinical trials showing an odds ratio of 0.47 (95% CI: 0.23–0.95) [39]. Of note, we did not find increased risk of hospitalization for heart failure with the use of DPP-4i, contrasting with the results of the EXamination of CArdiovascular OutcoMes with AlogliptIN versus Standard of CarE in Patients with Type 2 Diabetes Mellitus and Acute Coronary Syndrome trial [9] and the Saxagliptin Assessment of Vascular Outcomes Recorded in Patients with Diabetes Mellitus trial [10]. This may be due to restricting the study cohort to low-risk individuals.

Two observational studies using the Taiwan National Health Insurance Research Database also showed similar results to ours. The first of these from 2015 showed lower risk for MACE associated with DPP-4i compared to sulfonylurea as add-on therapy to metformin (aHR: 0.68, 95% CI: 0.55–0.83) [12]. Our study population expanded on this approach through the inclusion of individuals exposed to only metformin. Additionally, the Huang et al study of non-CKD patients showed lower risk of MACE among DPP-4i users than non-DPP-4i users (HR: 0.73, 95% CI: 0.61–0.87) [15]. Unlike this study however, we used negative (metformin) and positive (sulfonylurea) control groups instead of one control group with multiple drug classes. This allowed us to show that risk of MACE with DPP-4i was different when compared to high- and low-cardiovascular risk drug classes.

This analysis had two notable strengths. First, we used a low-risk population together with a new user design, allowing us to hone in on a common prescribing scenario of middle-aged patients without cardiovascular or renal disease initiating oral antihyperglycemic therapy, estimated to represent 69% of newly diagnosed patients [40]. Understanding whether or not diabetic treatment in this population increases patient risk for MACE is critical to managing their care due to the relationship between cardiovascular disease and diabetes [41]. Additionally, by restricting to a low baseline risk population, we were able to minimize potential confounding by indication due to prescribing practices for patients with underlying cardiovascular or renal disease.

Second, our study utilized a large, nationally representative dataset, allowing for a study population much larger than the clinical trials investigating the association of DPP-4i and MACE. All key trials examining any DPP-4i and cardiovascular outcomes had 53,769 altogether [42]. By using real-world data, we were able to assess the class-wide relationship of DPP-4i to MACE among patients with a variety of comorbidities and concomitant medications.

There were also limitations with our analysis. First, we allowed inclusion of individuals into more than one exposure group at baseline. We recognize that this could have potentially led to misclassification [43]. Excluding patients who were exposed to multiple drug groups of interest would have resulted in a 32.7% reduction in our population of individuals exposed to sulfonylurea, potentially threatening the generalizability of our results. As such, we assessed the sensitivity of our results to their inclusion and found them to be robust. The second limitation was our inability to account for time-varying hazards. We attempted to address this by introducing natural regression spline terms with knots at 6-, 12-, and 18-months of cumulative exposure. This allowed us to account for possible changes in baseline hazard with increasing cumulative exposure. Third, there is the potential for informative censoring due to a change in exposure status. We assessed sensitivity of our results to this approach to censoring and found them to be insensitive to the lagged latency period after the last day of exposure. Additionally, MACE primarily occurred within the first year of exposure and extending the latency period past 30-days would have unlikely changed our results and could have potentially led to misclassification.

Finally, due to dataset limitations and the observational nature of the study, we were unable to assess heterogeneity withing drug classes and key unmeasured variables. The first of these was mortality, which we could not include as a component of the primary composite outcome or as a competing risk. We also did not have data on body mass index, socio-economic status, or access to and quality of care, all correlates of cardiovascular disease. Additionally, we did not have sufficient data on duration of diabetes, basic metabolic panel results, blood pressure, smoking status, or glucose control. Our study population consisted of younger, low-risk patients with diabetes below age 65. As we were interested in those with low-cardiovascular risk, this younger cohort was representative of this patient subtype.

## Conclusion

Among commercially insured patients with diabetes and low-risk for MACE in the United States, our results provide evidence of decreased risk for MACE when comparing DPP-4i versus sulfonylurea. Additionally, we found that DPP-4i carried similar risk for MACE when compared to metformin. These results were also reflected in several individual components of the composite outcome, namely acute myocardial infarction, stroke, and heart failure. Finally, our results suggest that DPP-4i is a low cardiovascular risk option for low-risk patients initiating antihyperglycemic treatment. Further research is needed to investigate whether this association between DPP-4i and MACE is similar in high-risk populations.

## Supporting information

S1 Checklist STROBE statement—checklist of items that should be included in reports of *cohort studies*. (PDF)

S1 Table Categorization of drugs into exposure groups. List of all drugs classified in each exposure group.
(PDF)

S2 Table Covariates used in propensity score model and adjusted Cox proportional hazards model. List of covariates measured and included in the calculation of the propensity score and/or in the analysis as a covariate or stratifier.
(PDF)

S3 Table Hazard ratios for the association between DPP-4 inhibitor use and primary composite outcome, showing sensitivity to individuals with more than one exposure group. Sensitivity analysis to assess the robustness of primary and secondary outcomes to the addition of patients with more than one exposure.
(PDF)

S4 Table Hazard ratios for the association between DPP-4 inhibitor use and primary composite outcome, showing sensitivity to latency after drug discontinuation. Sensitivity analysis to assess the robustness of primary and secondary outcomes to the lagged latency periods after last dose of exposure dose.
(PDF)
