# Supplementary material for: Dipeptidyl peptidase-4 inhibitors and cardiovascular events in patients with type 2 diabetes, without cardiovascular or renal disease
Source: PLoS One. 2020 Oct 15;15(10):e0240141. doi: 10.1371/journal.pone.0240141 (PMC7561135; doi:10.1371/journal.pone.0240141)
Supplement: S1 Table — List of all drugs classified in each exposure group. (PDF) [file pone.0240141.s002.pdf]

Supplementary Material

S1 Table. Categorization of drugs into exposure groups

FDA Approved DPP-4 Inhibitors

- alogliptin
- alogliptin and metformin
- alogliptin and pioglitazone
- linagliptin
- linagliptin and empagliflozin
- linagliptin and metformin
- linagliptin and metformin extended release
- sitagliptin
- sitagliptin and metformin
- sitagliptin and metformin extended release
- saxagliptin
- saxagliptin and metformin extended release

FDA Approved Sulfonylureas

- chlorpropamide
- glimepiride
- glimepiride and pioglitazone hydrochloride
- glimepiride and rosiglitazone maleate
- glipizide
- glipizide extended release
- glyburide
- glyburide and metformin hydrochloride
- tolazamide
- tolbutamide

FDA Approved Metformin

- metformin hydrochloride
- metformin hydrochloride extended release
