# Supplementary material for: Dipeptidyl peptidase-4 inhibitors and cardiovascular events in patients with type 2 diabetes, without cardiovascular or renal disease
Source: PLoS One. 2020 Oct 15;15(10):e0240141. doi: 10.1371/journal.pone.0240141 (PMC7561135; doi:10.1371/journal.pone.0240141)
Supplement: S2 Table — List of covariates measured and included in the calculation of the propensity score and/or in the analysis as a covariate or stratifier. (PDF) [file pone.0240141.s003.pdf]

S2 Table. Covariates used in propensity score model and adjusted Cox proportional hazards model

Covariate	Measured	Included in Propensity Score Model	Included in adjusted Cox PH Model	
			Covariate	Stratifier
Demographics				
Male	X	X		X
Age	X	X		X
Location (Region)	X			
Cumulative Exposure	X			X
Comorbidities in Baseline				
Asthma	X	X		
Peripheral Vascular Disease	X	X		
Ischemic heart disease	X			
Hypertension	X	X	X	
Retinopathy	X	X		
Eye disease	X	X	X	
Renal disease	X		X	
Atrial fibrillation	X			
Neuropathy	X	X	X	
Nephropathy	X			
aDCSI Score	X			
Dual Exposures			X	
Sulfonylureas	X		X	
Metformin	X		X	
DPP-4 inhibitors	X			
Concomitant Medications at Baseline				
ACE inhibitors	X	X		
alpha agonists	X			
analgesics	X			
angiotensin II receptor blockers	X	X	X	
anti Veg-F	X			
anticoagulants	X			
antidepressants	X	X		
antiplatelets	X	X		X

S2 Table (cont'd). Covariates used in propensity score model and adjusted Cox proportional hazards model

Covariate	Measured	Included in Propensity Score Model	Included in adjusted Cox PH Model	
			Covariate	Stratifier
Concomitant Medications at Baseline				
aspirin	X			
benzodiazepines	X	X	X	
beta blockers	X	X		X
beta blockers (ophthalmic)	X			
bile acid sequestrants	X			
biologic response modifiers	X			
blood thinners and anticoagulants	X			X
bronchodilators	X	X		X
calcium channel blockers	X	X	X	
carbonic anhydrase inhibitors	X			
cardioselective beta blockers	X	X		
cholinergics	X			
disease-modifying antirheumatic drugs	X	X		
diuretics	X	X		
erythropoietin	X			
fibrates	X	X		
hormone replacement therapy	X	X		
inhaled steroids	X	X		X
leukotrine modifiers	X			
loop diuretics	X			
MAOI	X			
niacin	X			
nitrates	X			
NSAIDs	X	X		X
ophthalmic drugs	X			
oral corticosteroids	X	X		
other asthma medication	X			
peripheral neuropathic treatments	X	X	X	
phosphodiesterase-4 inhibitors	X			
potassium sparing diuretics	X			
prostaglandins	X			
SNRI	X			

S2 Table (cont'd). Covariates used in propensity score model and adjusted Cox proportional hazards model

Covariate	Measured	Included in Propensity Score Model	Included in adjusted Cox PH Model	
			Covariate	Stratifier
Concomitant Medications at Baseline				
SSRI	**X**			
statins	**X**	**X**	**X**	
theophyllines	**X**			
thiazide diuretics	**X**	**X**	**X**	
tricyclic antidepressants	**X**			
vasodilators	**X**			
α-Glucosidase inhibitors	**X**			
