# Supplementary material for: Dipeptidyl peptidase-4 inhibitors and cardiovascular events in patients with type 2 diabetes, without cardiovascular or renal disease
Source: PLoS One. 2020 Oct 15;15(10):e0240141. doi: 10.1371/journal.pone.0240141 (PMC7561135; doi:10.1371/journal.pone.0240141)
Supplement: S3 Table — Sensitivity analysis to assess the robustness of primary and secondary outcomes to the addition of patients with more than one exposure. (PDF) [file pone.0240141.s004.pdf]

S3 Table. Hazard ratios for the association between DPP-4 inhibitor use and primary composite outcome, showing sensitivity to individuals with more than one exposure group

	**Hazard Ratios for DPP-4 Inhibitors Use**	
**Reference Drug**	**Allowing for dual exposure**	**Disallowing dual exposure**
Sulfonylureas		
HR (95% CI)[1]	**0.86 [0.77, 0.97]**	**0.86 [0.76, 0.96]**
aHR (95% CI)[2]	**0.87 [0.78, 0.98]**	**0.87 [0.77, 0.97]**
Metformin		
HR (95% CI)[1]	1.08 [0.98, 1.19]	1.10 [1.00, 1.21]
aHR (95% CI)[2]	1.07 [0.97, 1.18]	1.10 [1.00, 1.21]

[1] Propensity score weighting only

[2] Propensity score weighting, spline terms for cumulative exposure, and demographics, comorbidities, and concomitant medications as regressors and stratifiers
